# Supplementary material for: Prevalence and characterisation of carbapenemase encoding genes in multidrug-resistant Gram-negative bacilli
Source: PLoS One. 2021 Nov 1;16(11):e0259005. doi: 10.1371/journal.pone.0259005 (PMC8559951; doi:10.1371/journal.pone.0259005)
Supplement: S1 Fig — (PDF) [file pone.0259005.s001.pdf]

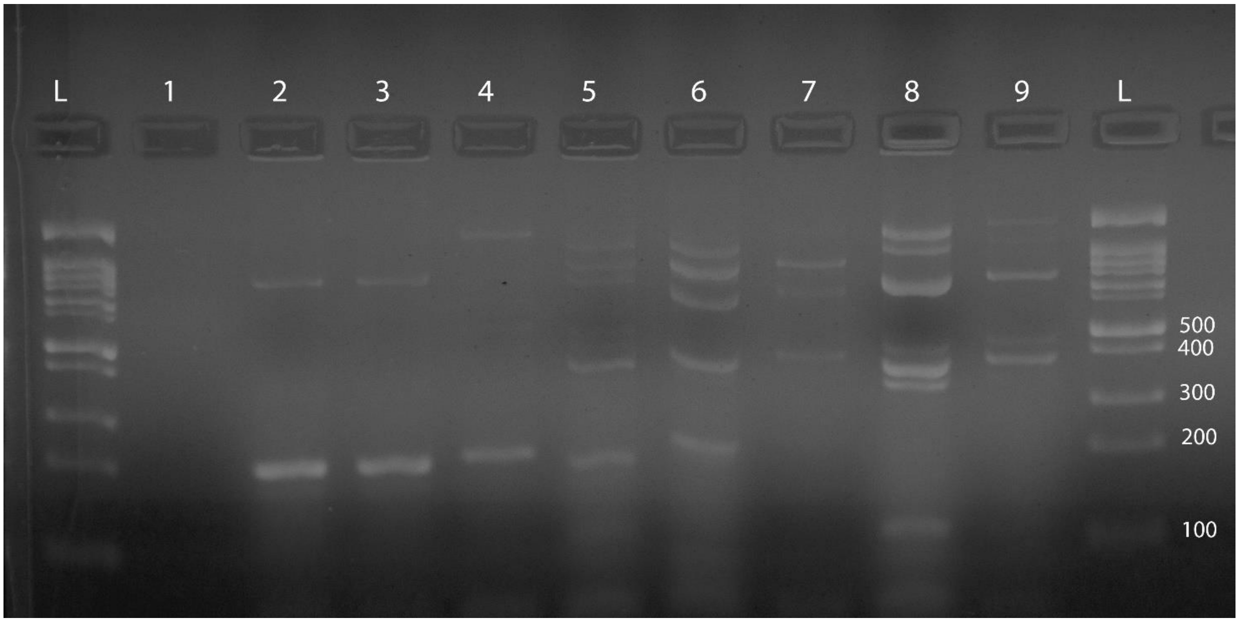

Detection of carbapenemase genes IMP, and VIM in carbapenemase-producing Gram-negative bacilli isolates, using multiplex PCR **(Original image)**

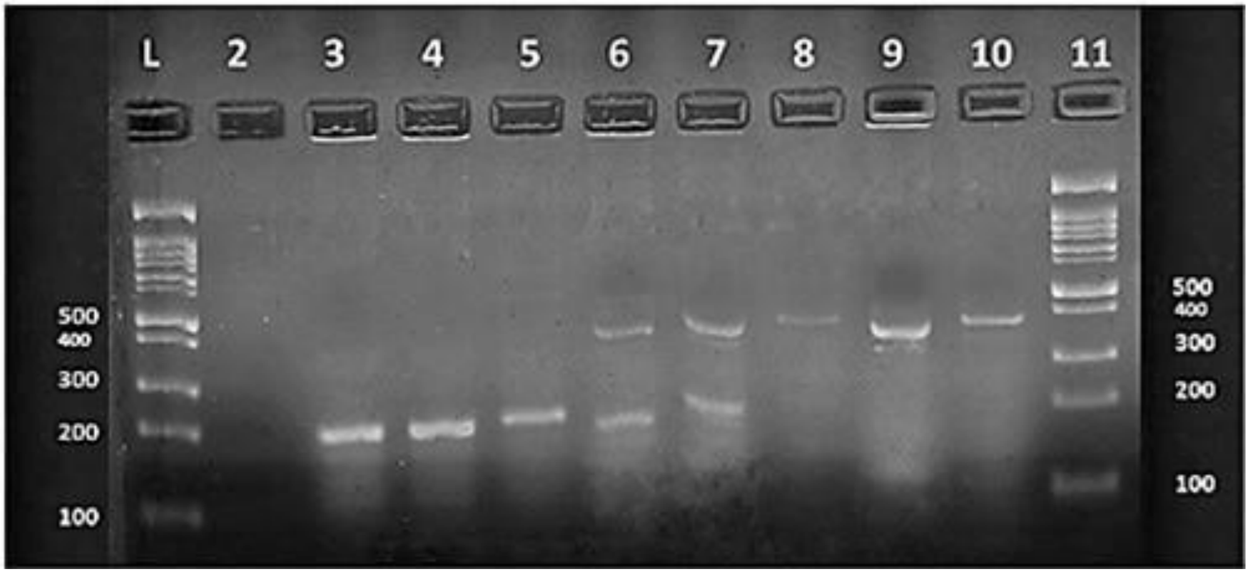

Fig 2.1. Detection of carbapenemase genes IMP, and VIM in carbapenemase-producing Gram-negative bacilli isolates, using multiplex PCR. Lanes 3–5 represent the positive IMP carbapenemase gene, Lanes 8–10 represent the positive VIM carbapenemase gene, Lanes 6 and 7 represent co-expression of IMP and VIM genes. The molecular size of the IMP and VIM genes are 232 and 390 bp respectively. Lane 1 and 11 are a 1-kb DNA ladder. Lane 2 corresponds to the negative control.

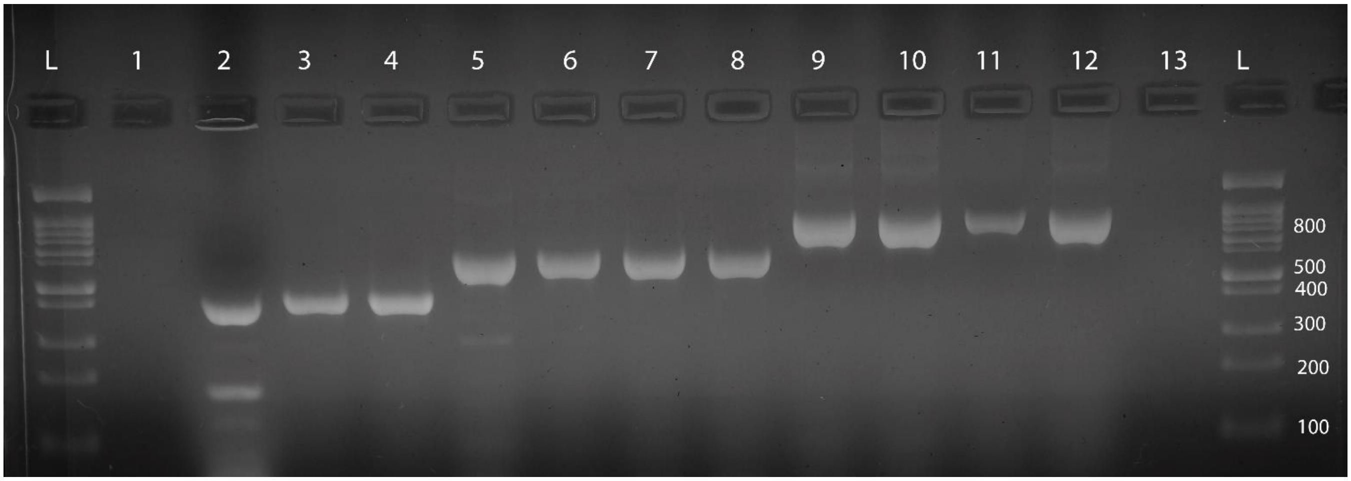

Detection of the carbapenemase genes OXA, NDM, and KPC in carbapenemase-producing Gram-negative bacilli isolates, using singleplex PCR **(Original image)**

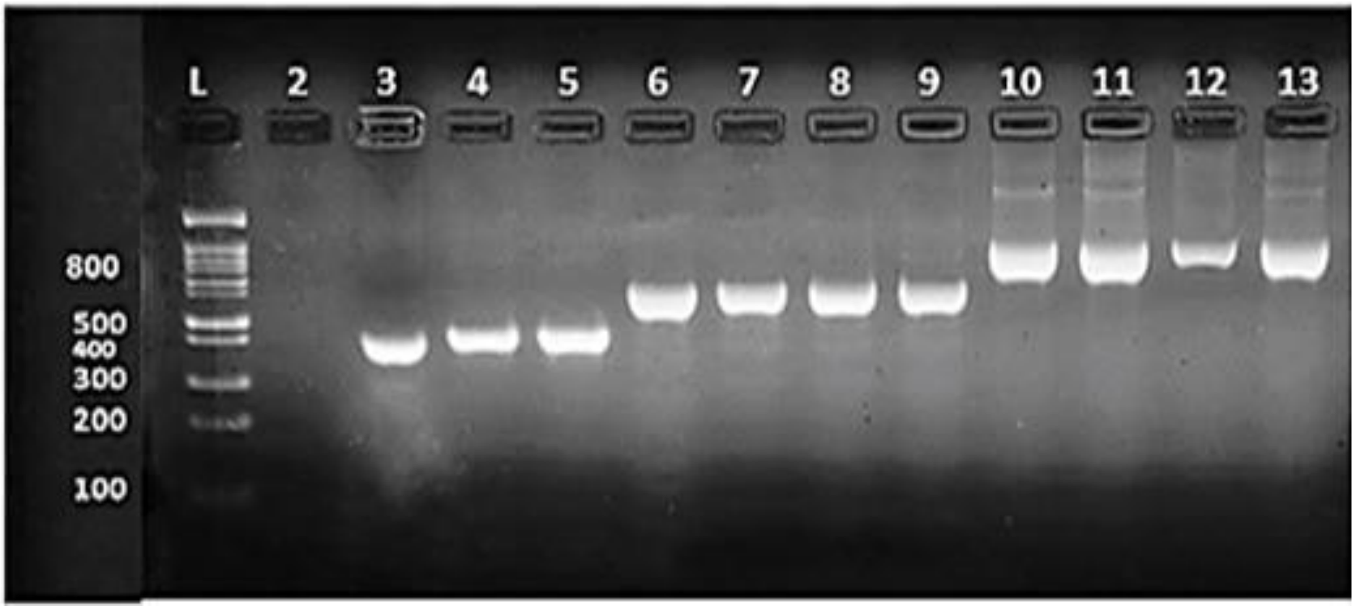

Fig 2.2. Detection of the carbapenemase genes OXA, NDM, and KPC in carbapenemase-producing Gram-negative bacilli isolates, using singleplex PCR. Lanes 3–5 represent the positive OXA-48 gene, Lanes 6–9 represent the positive NDM gene, and Lanes 10–13 represent the positive KPC gene. Lane 1 is a 1-kb DNA ladder. Lane 2 corresponds to the negative control. The molecular size of the tested *bla* $_{\text{OXA-48}}$, *bla* $_{\text{NDM}}$, and *bla* $_{\text{KPC}}$ carbapenemase genes are 438, 621 and 798 bp respectively.
