# Supplementary material for: Prevalence and characterisation of carbapenemase encoding genes in multidrug-resistant Gram-negative bacilli
Source: PLoS One. 2021 Nov 1;16(11):e0259005. doi: 10.1371/journal.pone.0259005 (PMC8559951; doi:10.1371/journal.pone.0259005)
Supplement: Attachment — Submitted filename: Response to Reviewers30.08.docx [file pone.0259005.s004.docx]

**Dear Dr. Abdelazeem**

Thank you for reviewing my manuscript. I appreciate your efforts and the efforts of the reviewers.

I have worked hard to edit the revised manuscript and I hope I have done well

I look forward to having my research accepted in PLOS ONE journal.

Sayran H. Haji

Author

**Journal Requirements:**

When submitting your revision, we need you to address these additional requirements.

1. Please ensure that your manuscript meets PLOS ONE's style requirements, including those for file naming. The PLOS ONE style templates can be found at
https://journals.plos.org/plosone/s/file?id=wjVg/PLOSOne_formatting_sample_main_body.pdf and
https://journals.plos.org/plosone/s/file?id=ba62/PLOSOne_formatting_sample_title_authors_affiliations.pdf

I tried to be in accordance with the requirements of PLOS ONE style

------------------------------------------------------------------------------------

2. In your Methods section, please provide additional information on the verbal consent provided by the patients, including as to how this verbal consent was documented or witnessed.

I added regarding oral consent in the ethical issues section

------------------------------------------------------------------------------------

3. PLOS ONE now requires that authors provide the original uncropped and unadjusted images underlying all blot or gel results reported in a submission’s figures or Supporting Information files. This policy and the journal’s other requirements for blot/gel reporting and figure preparation are described in detail at https://journals.plos.org/plosone/s/figures#loc-blot-and-gel-reporting-requirements and https://journals.plos.org/plosone/s/figures#loc-preparing-figures-from-image-files. When you submit your revised manuscript, please ensure that your figures adhere fully to these guidelines and provide the original underlying images for all blot or gel data reported in your submission. See the following link for instructions on providing the original image data: https://journals.plos.org/plosone/s/figures#loc-original-images-for-blots-and-gels.
 
In your cover letter, please note whether your blot/gel image data are in Supporting Information or posted at a public data repository, provide the repository URL if relevant, and provide specific details as to which raw blot/gel images, if any, are not available. Email us at plosone@plos.org if you have any questions.

My cover letter contains the DOI of the original, uncropped, unedited images of the blot /gel data published in a public data repository.

<https://data.mendeley.com/datasets/fhnkrf3jdr/1>

------------------------------------------------------------------------------------

4. In your Data Availability statement, you have not specified where the minimal data set underlying the results described in your manuscript can be found. PLOS defines a study's minimal data set as the underlying data used to reach the conclusions drawn in the manuscript and any additional data required to replicate the reported study findings in their entirety. All PLOS journals require that the minimal data set be made fully available. For more information about our data policy, please see http://journals.plos.org/plosone/s/data-availability.

Upon re-submitting your revised manuscript, please upload your study’s minimal underlying data set as either Supporting Information files or to a stable, public repository and include, or accession numbers within your revised cover letter. For a list of acceptable repositories, please see http://journals.plos.org/plosone/s/data-availability#loc-recommended-repositories. Any potentially identifying patient information must be fully anonymized.

The minimal data set is available in public repository and include the relevant DOIs, within my revised cover letter.

Dataset: <https://data.mendeley.com/datasets/dp7g8rntyb/1>

Important: If there are ethical or legal restrictions to sharing your data publicly, please explain these restrictions in detail. Please see our guidelines for more information on what we consider unacceptable restrictions to publicly sharing data: http://journals.plos.org/plosone/s/data-availability#loc-unacceptable-data-access-restrictions. Note that it is not acceptable for the authors to be the sole named individuals responsible for ensuring data access.

I don’t have any ethical or legal restrictions

We will update your Data Availability statement to reflect the information you provide in your cover letter.

------------------------------------------------------------------------------------

5. Please ensure that you refer to Figure 1 in your text as, if accepted, production will need this reference to link the reader to the figure.

I referenced all the Figures in my text to link the reader to the figure.

------------------------------------------------------------------------------------

6. We note you have included a table to which you do not refer in the text of your manuscript. Please ensure that you refer to Table 1 in your text; if accepted, production will need this reference to link the reader to the Table.

I referenced all the tables in my script to connect the reader to the figure.
------------------------------------------------------------------------------------

Reviewer #1: Comments to authors:
- The current study is very interesting; however, the authors should address the following comments to improve the quality of the manuscript:
- The manuscript should be revised for language editing and grammar mistakes by a native English speaker.

I have checked for language editing and grammatical errors by native British speakers using online proofreading;

Professional editing and proofreading
services at your fingertips

<https://app.proofreadmyessay.co.uk/my-documents/>

------------------------------------------------------------------------------------

- Please write the genes name in correct form all over the manuscript (the 1st 3 letters must be small and italic and the other 3 letter are capital and subscripted)

I corrected the name of the genes throughout the manuscript

------------------------------------------------------------------------------------

Title:
I think the work would benefit from the title that contains main conclusion of the study (should be derived from the conclusion), please modify the title.

I modified the title that contains main conclusion

------------------------------------------------------------------------------------

Abstract:
- The abstract must illustrates the used methods and the most prevalent results (give more hints about methods and results). Besides, rephrase the main conclusion of your findings.

More hints were given about the methods and results. Besides, I reformulated the main conclusion of the results.
------------------------------------------------------------------------------------

Introduction:

-Give a hint about different infections caused by common pathogenic members of Enterobacteriaceae (such as E. coli and K. pneumonia) and Pseudomonas aeruginosa, their virulence factors, and the mechanism of disease occurrence.

Hints were given about the common pathogenic members of Enterobacteriaceae in the introduction part
------------------------------------------------------------------------------------

- The introduction needs to be more informative:
-The authors should illustrate the public heath importance concerning the emergence of multidrug-resistant (MDR) bacterial pathogens that reflecting the necessary of new potent and safe antimicrobial agents. Several studies proved the widespread MDR- bacterial pathogens;
Authors could add the following paragraph:
Multidrug resistance has been increased globally that is considered public health threat. Several recent studies reported the existence of multidrug-resistant bacterial pathogens from different origins including humans, poultry, cattle, and fish that increase the need for routine application of the antimicrobial susceptibility testing to detect the antibiotic of choice as well as screening of the emerging MDR strains. You should cite the following valuable studies:
1-PMID: 33177849
2-PMID: 32497922
3-PMID:33061472
4-PMID: 33947875
5-PMID: 32472209
6-PMID: 32994450

I have illustrated the public health importance of the emergence of multidrug-resistant (MDR) bacterial pathogens by adding your paragraph, and I really appreciate your help in providing these valuable studies, which I used as references.

------------------------------------------------------------------------------------

-Rephrase the aim of work to be clear and better sound.

I paraphrased the aim of the work

Material and methods
-Table 1 must be placed in the results section.

I have moved the table to the resulting part
------------------------------------------------------------------------------------

-It is not acceptable to illustrate the prevalence of the bacterial pathogens in the methods; it should be placed to the results section. I have moved the prevalence of bacterial pathogens to the results section.
------------------------------------------------------------------------------------

-Where are the methods of the isolation and identification of 200 Gram-negative bacteria?? You must illustrate in details and support your methods with specific references.

The methods for isolating and identifying 200 Gram-negative bacteria have been explained with adding specific references. Furthermore, I added a new table to identify 200 Gram-negative bacteria.
-----------------------------------------------------------------------------------

- Phenotype-based method for carbapenemases: should be modified to be: Phenotypedetection carbapenemases using Disc diffusion method. Rephrase this section; add more details, discuss in details and specific references.

I have modified the phenotypic-based method for carbapenemes to the phenotype detection of carbapenemes using the disc diffusion method, and more details have been added to this section with references.

------------------------------------------------------------------------------------

- Confirmation of carbapenemase production using commercial
combination disk assay; add specific references to this section.

The phenotypic-based method has been combined with confirmation of carbapenemase production using commercial combination disk checked, because I think it was duplicated, and specific references were added

------------------------------------------------------------------------------------

-Molecular detection of genes should be modified:
PCR-based detection of carbapenemase genes. Besides, add specific references.

Molecular gene detection was changed to PCR-based detection of carbapenemase genes. Besides, specific references have been added.

------------------------------------------------------------------------------------

-Where are the statistical analyses?

Statistical analyzes for this study were designed using GraphPad Prism (version 5; GraphPad Software, San Diego, CA). The Chi-square test was applied to analyze the obtained data.

------------------------------------------------------------------------------------

-Results:
- Where are the results of phenotypic characters and the prevalence of the isolated bacterial pathogens from the examined samples? You must illustrate their prevalence in a new table.

The results of the phenotypic characteristics and prevalence of bacterial pathogens isolated from the examined samples were added to a new table with a clear explanation of the correlation in the text. ------------------------------------------------------------------------------------

-Table 1 must be placed in the results section.

Table 1 has been converted to Table 2 and moved to the result section.

-----------------------------------------------------------------------------------

- Illustrate in a new table the test results of the phenotypic MDR (illustrate the antimicrobial classes of the used antimicrobial agents).

I have developed a new table of MDR phenotypic test results identifying the antimicrobial classes of the antimicrobial agents used.

------------------------------------------------------------------------------------

-Illustrate the distribution of the carbapenemase genes among the recovered isolates in a new table.
The distribution of carbapenemase genes among the recovered isolates is illustrated in a new table

------------------------------------------------------------------------------------

- Please increase the resolution of all figures (1200 dpi).

I have increased the resolution. Hope it is clear now

------------------------------------------------------------------------------------

-Where are the statistical analyses?

Statistical analyzes for this study were designed using GraphPad Prism (version 5; GraphPad Software, San Diego, CA). The Chi-square test was applied to analyze the obtained data.

------------------------------------------------------------------------------------

-Discussion:
-The discussion is too long and poor; the authors are advised to illustrate the real impact of their findings without repetition of results.

I've shortened the discussion and deleted all the duplicates as much as I can

------------------------------------------------------------------------------------

-Conclusion
- Should be rephrased to be sounded. A real conclusion should focus on the question or claim you articulated in your study, whose resolution has been the main objective of your paper?

The conclusion has been reformulated to be presented and includes the main resolution of my study.

------------------------------------------------------------------------------------

Reviewer #2: - The current study has a significant impact, but it needs a major revision:
- The manuscript should be revised for grammar mistakes.

I have checked for language editing and grammatical errors by native British speakers using online proofreading;

Professional editing and proofreading
services at your fingertips

https://app.proofreadmyessay.co.uk/my-documents/

------------------------------------------------------------------------------------

-The title is broad, please modify the title.

I have modified and shortened the title

------------------------------------------------------------------------------------

-In the introduction: discuss the public health importance of the members of Enterobacteriaceae and other Gram-negative bacterial pathogens.

The public health importance of the members of Enterobacteriaceae and other Gram-negative bacterial pathogens was discussed in the introduction part

------------------------------------------------------------------------------------

-Improve the aim of work.

The purpose of the work has improved
------------------------------------------------------------------------------------

Methods:
-Transfer Table 1 to the results.

I have moved the table to the resulting part

------------------------------------------------------------------------------------

-Transfer the prevalence of bacterial species to the results section.

I have moved the prevalence of bacterial pathogens to the results section.
------------------------------------------------------------------------------------

-Discuss the isolation and identification of the bacterial pathogens.

The methods for isolating and identifying Gram-negative bacteria have been explained with adding specific references. Furthermore, I added a new table to identify 200 Gram-negative bacteria.

------------------------------------------------------------------------------------

-Specific references should be added to all the used methods and techniques.

I added specific references to all the methods and techniques used

------------------------------------------------------------------------------------

-Where is the statistical analysis?

Statistical analyzes for this study were designed using GraphPad Prism (version 5; GraphPad Software, San Diego, CA). The Chi-square test was applied to analyze the obtained data.

------------------------------------------------------------------------------------

-Results:
- Discuss the prevalence of the retrieved isolates in a new table.

The results of the prevalence of bacterial pathogens isolated from the examined samples were added to a new table with a clear explanation in the text.

------------------------------------------------------------------------------------

- Transfer Table 1 to the results

Table 1 has been converted to Table 2 and moved to the result section.

------------------------------------------------------------------------------------

- Please increase the resolution of all figures (1200 dpi).

I have increased the resolution. Hope it is clear now

-----------------------------------------------------------------------------------

-Discussion:
-Very long; please improve

I've shortened and improved the discussion as much as I can
