# Supplementary material for: Prevalence and characterisation of carbapenemase encoding genes in multidrug-resistant Gram-negative bacilli
Source: PLoS One. 2021 Nov 1;16(11):e0259005. doi: 10.1371/journal.pone.0259005 (PMC8559951; doi:10.1371/journal.pone.0259005)
Supplement: Attachment — Submitted filename: Response to Reviewers.docx [file pone.0259005.s006.docx]

**Dear Dr. Abdelazeem**

Thank you for reviewing my manuscript. I appreciate your efforts and the efforts of the reviewers.

I really apologize for my mistakes it wasn't intentional

I don't want to lose my chance of getting acceptance in PLOS ONE journal I did my best

Please help me if I'm wrong about something

I look forward to having my research accepted in PLOS ONE journal.

Sayran H. Haji

Author

**Journal Requirements:**

Reviewer #1: In the introduction section the authors reported several recent studies in the following paragraph,
however they have cited only one reference.. How??
Please cite all references:

With my appreciation, I have referred to all of the following valuable studies as references in the introduction, material methods, and results in the manuscript.
1-PMID: 33177849	reference number 30
2-PMID:33061472	reference number 3
3-PMID: 33947875	reference number 1

4-PMID: 32472209	reference number 22
5-PMID: 32994450	reference number 2

Lines 438- 505; please add specific references. The following sentence is found instead of refrences; please revise and correct. Error! Reference source not found.

I have revised and corrected the error related to the reference source on lines 438-505.
